# Winding number selection on merons by Gaussian curvature’s sign

**DOI:** 10.1038/s41598-019-50395-7

**Published:** 2019-10-04

**Authors:** Ricardo Gabriel Elías, Nicolás Vidal-Silva, Vagson L. Carvalho-Santos

**Affiliations:** 1 Departamento de Física, Universidad de Santiago de Chile, Avda. Ecuador, 3493 Santiago, Chile; 2 CEDENNA, Universidad de Santiago de Chile, Avda. Ecuador, 3493 Santiago, Chile; 3 Departamento de Física, FCFM, Universidad de Chile, Santiago, Chile; 4 Departamento de Física, Universidade Federal de Viçosa, Viçosa, Brazil

**Keywords:** Physics, Condensed-matter physics, Ferromagnetism

## Abstract

We study the relationship between the winding number of magnetic merons and the Gaussian curvature of two-dimensional magnetic surfaces. We show that positive (negative) Gaussian curvatures privilege merons with positive (negative) winding number. As in the case of unidimensional domain walls, we found that chirality is connected to the polarity of the core. Both effects allow to predict the topological properties of metastable states knowing the geometry of the surface. These features are related with the recently predicted Dzyaloshinskii-Moriya emergent term of curved surfaces. The presented results are at our knowledge the first ones drawing attention about a direct relation between geometric properties of the surfaces and the topology of the hosted solitons.

## Introduction

The relation between the curvature of materials and the topology of the configurations appearing on them is not completely understood neither mathematically nor physically. However, the study of these two concepts is a very old and established topic, as it can be seen for example in the Gauss-Bonnet theorem, which shows a relation between curvature and the Euler characteristic^1^. The curvature on two-dimensional condensed matter systems has consequences on the energetic properties and dynamics of quasiparticles and so also on the topology of the observed configurations. The changes in the topological charges of the configurations minimizing the energy and the relation between geometry and topology has been addressed in many physical contexts^2^ as thin layers^3^, soft matter^4^, superfluid^5^, superconductor spheres^6^, superconductors tubes^7^, nematic liquid crystals^8^ and many others, including cell membranes^9^, graphene^10^ and topological insulators^11^.

In the context of magnetic materials, the effects of curvature (see^12^ for a review) on magnetochirality^13^, the formulation of exchange energy in terms of Gaussian and mean curvatures^14^ and some specific problems have been addressed: magnetochiral effects in tubular structures and the possibility to sustain stable domain walls (DWs) at large and constant propagation speed^15^, the geometric frustration in an infinite elastic cylinder^16^, the curvature of a Möbius ring, in which the topology of the ring favours the appearance of a DW and curvature is responsible for a Dzyaloshinskii-Moriya (DM) like term^17^ which couples the chiralities of the Möbius ring and magnetization, and the effects of the torsion and curvature on spin waves in nanowires^18^.

In the specific case of vortices, it has been shown that on the surface of a sphere its chirality is polarity-dependent (a phenomenon not observed in planar nanomagnets)^19^, which is actually a consequence of the interplay between the topology of the magnetic quasiparticle and the curvature of the surface. This curvature-induced polarity is also observed in domain walls hosted in curved nanowires^17,20^. Here we define the chirality *γ* of a meron type texture as the direction in which the magnetization is oriented respect to a radial direction defined on the surface. In this sense, a radial vortex^21^ has zero chirality while a tangent circulating one has chirality *π*/2 as is explicitly defined in Eq. (8).

A recent article describing the possibility of the development of vortex-antivortex pairs in magnetic toroidal shells^22^ has shown that the sign of the curvature could have an interesting effect on the preferred solitons to be hosted. In fact, regions of negative curvature prefer to accommodate anti-vortices while vortices are stabilized on positive curvature regions. In this sense, we explore the influence of the sign of the curvature on the local minima of the meron’s magnetic energy, composed by exchange and anisotropy terms. Meron is a generic name for vortices and anti-vortices and in general, they are half-integer topological charge structures. In this work, we find a suitable parametrization of a two-dimensional surface which allows us to change the curvature continuously exploring the negative and positive curvatures regions, and explore the energy of different magnetic configurations: vortices and anti-vortices with different chiralities and polarities, as well as the normal homogenous state.

This paper is organized as follows: In Section II, we introduce the physical model and the parametrization of a surface that goes continually from a paraboloid to a hyperbolic paraboloid depending on a continuous real parameter. In Section III we numerically calculate the energy for the different cases and present the discussion of the obtained results. Finally, in Section IV we present the conclusions and prospects.

## Magnetization over Paraboloid and Hyperbolic Paraboloid Surfaces

In general, given a two-dimensional surface parameterised by ***r*** = ***r***(*u*, *v*), where (*u*, *v*) are local curvilinear coordinates, the tangent vectors to the surfaces can be found by ***g***_*u*_ = ∂_*u*_***r*** and ***g***_*v*_ = ∂_*v*_***r***. From them, the elements *g*_*μν*_ of the metric tensor ***g*** are defined as ${g}_{\mu \nu }={{\boldsymbol{g}}}_{\mu }\cdot {{\boldsymbol{g}}}_{\nu }$ (for *μ*, *ν* = *u*, *v*). The inverse of the metric tensor is defined as *g*^*μα*^*g*_*αν*_ = ${\delta }_{\nu }^{\mu }$, where ${\delta }_{\nu }^{\mu }$ is the Kronecker delta and where repeated indices are implicitly summed over. With this notation, the energy of a two-dimensional ferromagnetic material with normalized order parameter ***m***(***r***, *t*) with exchange and anisotropy energies is given by

1 $${E}_{{\rm{tot}}}=A\int [{g}^{\mu \nu }{\partial }_{\mu }{\boldsymbol{m}}\cdot {\partial }_{\nu }{\boldsymbol{m}}+\frac{{({\boldsymbol{m}}\cdot {\boldsymbol{n}})}^{2}}{{\ell }^{2}}]\sqrt{g}\,du\,dv,$$

where *g* = det ***g***, *A* is the exchange constant and the magnetic length is defined as $\ell =\sqrt{A/{K}_{a}}$, where *K*_*a*_ > 0 is the anisotropy constant and we are considering an in-surface anisotropy. The vector ***n*** is the normal vector to the surface.

**Figure 1:**
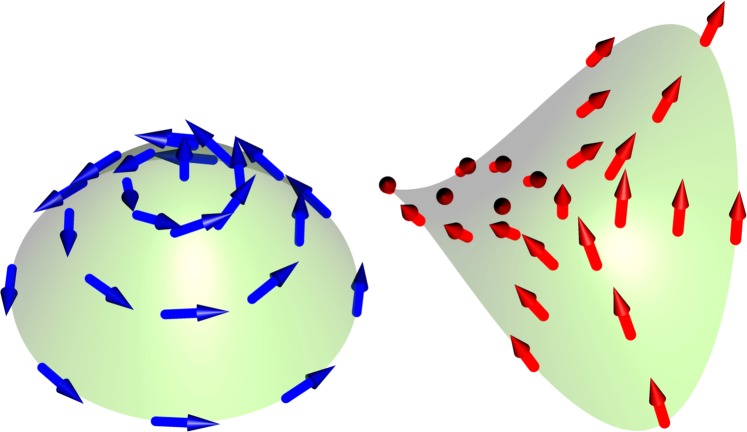
Considered magnetic surfaces and magnetic configurations minimizing the energy. Paraboloid (*c* = 1), hyperbolic paraboloid (*c* = −1). We have depicted a magnetic vortex (blue arrows) on the paraboloid and an antivortex (red arrows) on the hyperbolic paraboloid.

In order to explore the relation between the curvature of the surface and the energy of different magnetic configurations, the considered magnetic system will be a surface whose shape depends on an adimensional real parameter *c* ∈ [−1, 1], parameterized by

2 $${\boldsymbol{r}}=(x,y,c(c{x}^{2}+{y}^{2})),$$

where (*x*, *y*, *z*) are the cartesian coordinates of the three-dimensional space. The *c* parameter controls the curvature of the surface going continuously from a hyperbolic paraboloid (*c* = −1) to a paraboloid (*c* = 1) and passing by a flat surface (*c* = 0) (see Fig. 1), respectively. We have inserted a global *c* factor in the *z* coordinate in order to impose the flat surface when *c* = 0 (otherwise, we would obtain a cylinder geometry at *c* = 0). The Gaussian curvature can be readily calculated from the metric tensor ***g*** (or the closely related first fundamental form)

3 $${\boldsymbol{g}}=(\begin{array}{ll}E & F\\ F & G\end{array})=(\begin{array}{ll}1+4{c}^{4}{x}^{2} & 4{c}^{3}xy\\ 4{c}^{3}xy & 1+4{c}^{2}{y}^{2}\end{array})$$

and the second fundamental form^1^, that in this case has the components *L* = *N* = 2*c*^2^ and *M* = 0. The Gaussian curvature (just curvature from now on) is then given *K* = (*LN* − *M*^2^)/(*EG* − *F*^2^), explicitly written as

4 $$K=\frac{4{c}^{3}}{1+4{c}^{2}({c}^{2}{x}^{2}+{y}^{2})}.$$

This curvature is anti-symmetrical in *c* (and it is axis-symmetrical for *c* = ±1). Therefore, it allows us to analyse the changes in sign of the curvature regardless of the specific details of the surface and comparing the opposite sign case.

In this work, we are mainly interested in analising the energy of merons. The description of the magnetization of vortices and antivortices in this geometry will be done by using the local coordinates on the surface ***n***_*ρ*_ = ***g***_*ρ*_/||***g***_*ρ*_||, ***n***_*ϕ*_ = ***g***_*ϕ*_/||***g***_*ϕ*_|| and the normal vector to the surface ***n*** = ***n***_*ρ*_ × ***n***_*ϕ*_/||***n***_*ρ*_ × ***n***_*ϕ*_|| (we need to normalize this last product because of the non-diagonal behaviour of ***g***). In the above definitions we use polar coordinates in the plane $\rho =\sqrt{{x}^{2}+{y}^{2}}$ and *ϕ* = arctan(*y*/*x*). Note that with this choice of coordinates the cases *c* = 0 and *c* = 1 present now a diagonal metric, while the case *c* = −1 keeps a non-diagonal metric.

From these definitions a vector field on the surface can be written as

5 $${\boldsymbol{N}}({\boldsymbol{r}},t)=\,\cos \,\Phi \,\sin \,\Theta {{\boldsymbol{n}}}_{\rho }+\,\sin \,\Phi \,\sin \,\Theta {{\boldsymbol{n}}}_{\varphi }+\,\cos \,\Theta {\boldsymbol{n}}.$$

where Θ = Θ(***r***, *t*) and Φ = Φ(***r***, *t*) are the local spherical field coordinates in which the angles Θ and Φ are the angles with respect to the normal and the angle of the projection of the field onto the tangent plane with the ***n***_*ρ*_ tangent vector, respectively. Then, we define the normalized magnetization as

6 $${\boldsymbol{m}}({\boldsymbol{r}},t)=\frac{{\boldsymbol{N}}({\boldsymbol{r}},t)}{||{\boldsymbol{N}}({\boldsymbol{r}},t)||},$$

with $||{\boldsymbol{N}}({\boldsymbol{r}},t)||=\sqrt{1+\,\sin (2\Phi )({\cos }^{2}\Theta ){{\boldsymbol{n}}}_{\rho }\cdot {{\boldsymbol{n}}}_{\varphi }}$. The formal description of a meron solution will be given by the ansatz^23^

7 $$\cos \,\Theta (\rho )=\frac{p}{1+{(\frac{\rho }{{r}_{0}})}^{s}}$$

8 $$\Phi (\varphi )=(q-1)\varphi +\gamma .$$

In these formulae, *q* is an integer number called the winding number of the structure and representing the curl of the field around the meron’s core when projecting the field onto the surface. In the simplest case, a vortex structure, we have that *q* = 1, while an antivortex is given by *q* = −1. In Eq. (8) *γ* determines the chirality of the meron, and it consists of a phase that gives the orientation of the field with respect to the radial direction on the surface. The parameter *s* is a positive integer that controls the size of the region between the core (with radius *r*_0_) and the in-surface regions. Finally, *p* is the polarity of the core, which can be 1 (parallel to the normal to the surface) or −1 (antiparallel to the normal). The topological charge is defined using the polarity and the winding number as *Q* = *pq*/2 in the case of merons and *Q* = *pq* in the skyrmion case^24,25^. It can be notice that in the adopted parametrization, a vortex (antivortex) is defined as a configuration that lies asymptotically in the in-surface plane. Due to the deformation of the surface, the radial direction in the surface ***n***_*ρ*_ direction is not necessarily orthogonal to ***n***_*ϕ*_. In this work, our discussion will focus mainly on merons, but our results can also be qualitatively extended to skyrmions.

## Results

### Relation between core and chirality

Following some recent results on nanowires^20^, we numerically explore the relation between the polarity of the core and the chirality of merons in its vicinity. In the cited work, the authors obtained that due to the appearing of a DM-like emergent field, as a consequence of curvature^12,14,21^, there is a selection in the phase on the azimuthal direction of a domain wall. Indeed, a head-to-head domain wall prefers pointing outward the bent wire, as long as a tail-to-tail domain wall points inward the bent wire.

In general, in two-dimensional magnets, the appearence of a DM-like term can be understood by writing the exchange energy in curved surfaces with diagonal metric as the addition of three terms^14,26^: the first one having the same structure of the exchange energy in a flat surface, the second one having the role of an anisotropy energy and the third one being first order in derivatives as in the usual DM interaction. Explicitly, the DM-like term can be written as^26^

9 $${E}_{{\rm{DM}}}=2{D}_{\alpha \beta \gamma }{m}_{\beta }{\partial }_{\gamma }{m}_{\alpha },$$

where the effective DM-like coefficients are

$${D}_{\alpha \beta \gamma }={{\boldsymbol{e}}}_{\alpha }\cdot \frac{{\partial }_{\gamma }}{\sqrt{{g}_{\gamma \gamma }}}{{\boldsymbol{e}}}_{\beta }$$

where ***e***_*μ*_ are the unitary orthogonal tangent vectors to the surface. Let’s note that because of ***e***_*α*_ ⋅ ***e***_*β*_ = 0 the *D*_*αβγ*_ is antisymmetric in *α*, *β*. These important results can be also applied to the understanding of a non-diagonal two-dimensional metric considering the fact that any two-dimensional Riemannian manifold can be described locally by a diagonal metric (see^27^ for more details). In this sense, even considering our metric is not diagonal, it can be locally diagonalized and the physical effects coming DM-like energy must remain.

**Figure 2:**
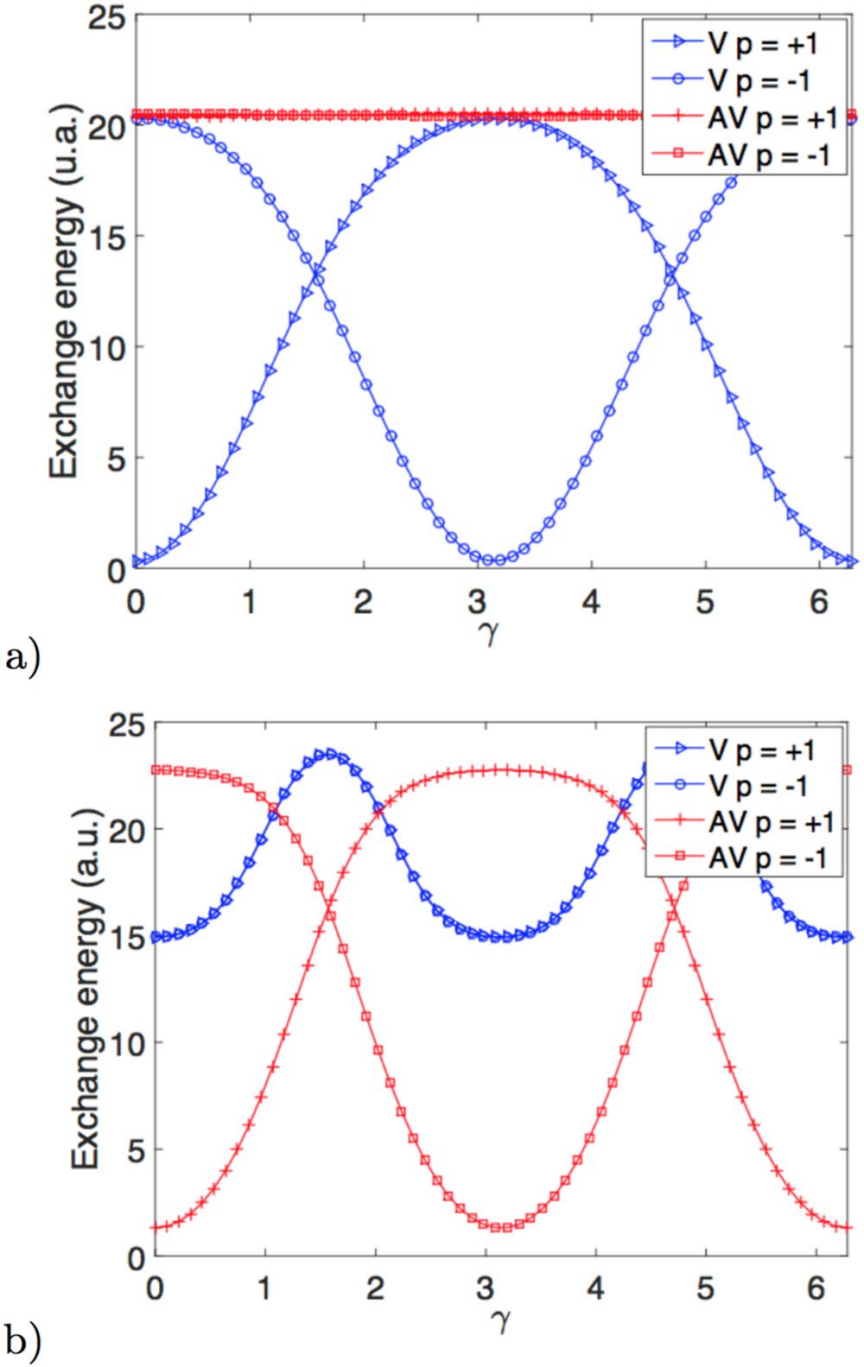
Exchange energy of vortex (blue) and antivortex (red) configurations in function of the chirality *γ*. Panel a) shows the paraboloid case *c* = 1 and panel b) presents hyperbolic paraboloid case *c* = −1.

In order to explore these ideas on a two-dimensional magnet in which there is a non-trivial topological defect, we use the ansatz given in Eq. (8) considering different winding numbers *q* and chiralities in surfaces with different gaussian curvature sign as in the two surfaces shown in Fig. 1. For all the calculations we have considered *s* = 2. We have observed (see Fig. 2) that, similarly to the results presented in ref.^20^, the polarity of the meron drastically changes the chirality that minimizes the exchange energy, evidencing the appearance of chiral effects in curved surfaces (let’s remind that *γ* is undetermined by the exchange energy in flat surfaces). Figure 2a shows explicitly the exchange energy of merons in a surface with positive curvature (*c* = 1). It is observed that for a vortex with positive polarity, the chirality minimizing the energy is *γ* = 0, and a head-to-head structure holds. On the other hand, a vortex with negative polarity presents the minimum exchange energy for *γ* = *π*, creating a tail-to-tail structure. Both results are in agreement with the findings on domain walls on curved wires^20^. Still in 2a) it can be noticed that the energy remains invariant as a function of *γ* for the anti-vortex case. This finding is coherent with the fact that changes in chirality are equivalent to rotations when we have an antivortex in an axisymmetric geometry.

In the *c* = −1 case, Fig. 2b, the situation is similar to the above case. The global minimum appears for the anti-vortex configuration with *γ* = 0 when *p* = 1 and *γ* = *π* when *p* = −1, which is again a manifestation of a curvature-induced polarity for a tail-to-tail and head-to-head magnetization configuration, correspondingly. Figure 2b) also shows that vortices have the same preferred chirality for both polarities, although these are energetically less favorable that the minima of antivortices.

The polarity-chirality coupling on magnetic solitons has been pointed out lately by some authors^12,14,21^, which shows that the curvature effects on the magnetic properties give rise to an effective DM interaction, responsible for these magnetochiral effects. Our results follow this same line, showing the same effects for non-diagonal metrics as is the case with *c* = −1.

### Relation between curvature and winding number

**Figure 3:**
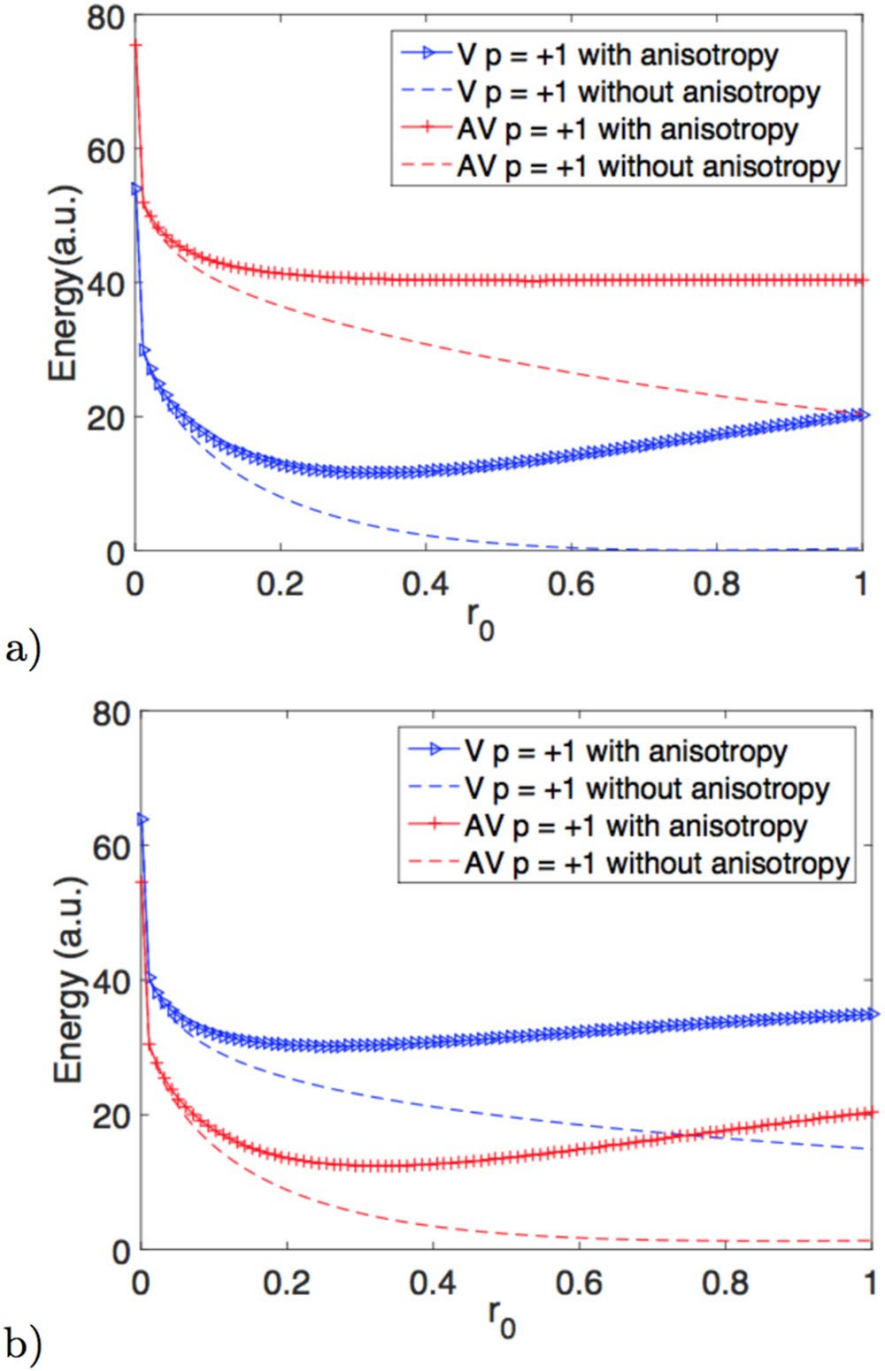
Total energy as a function of the core *r*_0_ of the meron for *K*_*a*_ = 5 (continuous lines) and *K*_*a*_ = 0 (dashed lines). In panel a) panel we show the paraboloid case and in the left one the hyperbolic paraboloid case. Both cases are depicted for *γ* = 0 and positive polarity, according to the results of Fig. 2.

Once understood the relation between polarity and chirality, we address the problem of the properties of meron’s core (which is roughly the interior region of the surface bounded by *ρ* = *r*_0_). In order to exclude the influence of the core’s size on the meron-like groundstate of the system, we include an easy-surface anisotropy term into the energy. It is worth to notice that in the usual flat case in the presence of DM interaction there is a competition between the DM parameter, the anisotropy energy and the exchange one which determines the size of the core^28,29^. In the model here considered, the DM-like is an emergent field coming from the exchange interaction on curved surfaces and it cannot be controlled independently, thus the only mechanism allowing a choice in the core size is the competition between anisotropy and exchange. As expected, the introduction of the anisotropy imposes a specific *r*_0_ which minimizes the total energy (see Fig. 3). Indeed, a divergence in the exchange energy related with the singularity in the azimuthal angle favors the formation of the meron’s core. However, the competition between exchange and anisotropy imposes that this core must have a specific radius *r*_0_ given by the interplay between anisotropy and exchange magnetic parameters of the materials. In this context, given an anisotropy and meron’s polarity, we can determine the values of *r*_0_ and *γ* minimizing the magnetic energy. It can be concluded from Fig. 3 that the global minimum of the energy is not determined by the meron’s core energy. That is, surfaces with positive curvatures present vortices while surfaces with negative curvatures present antivortices as meron-like groundstate.

**Figure 4:**
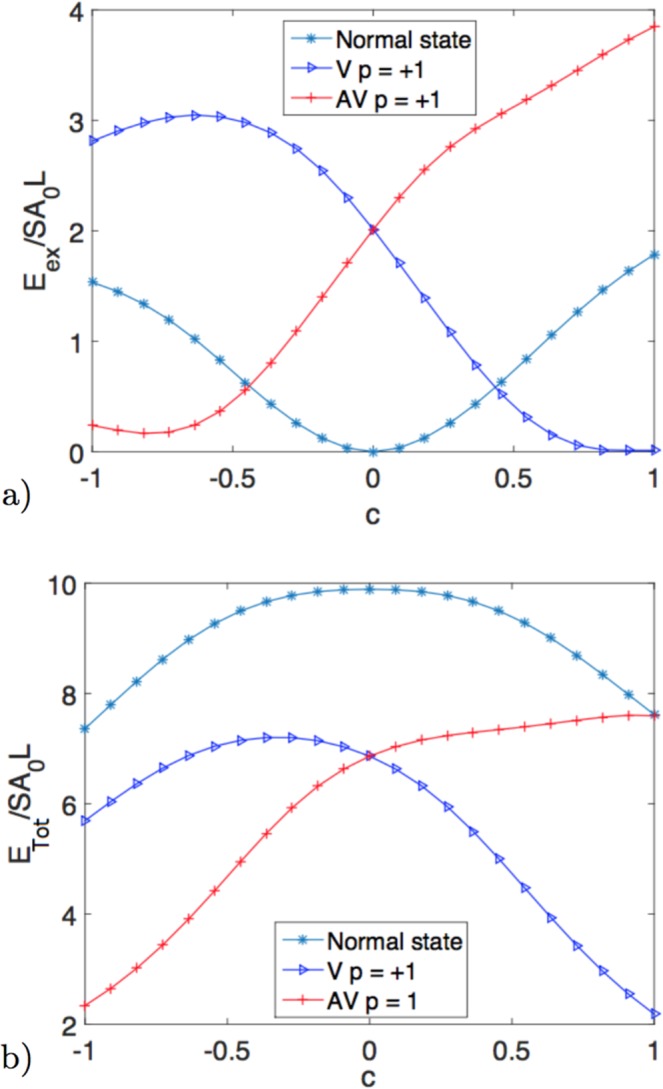
(**a**) Minimized exchange energy and (**b**) minimized total energy (exchange plus anisotropy) by unit surface for the vortex state (blue), the antivortex (red) and the normal configuration (light blue) as a function of the *c* parameter controlling the sign of the Gaussian curvature. Both merons are plotted for *γ* = 0 and *p* = 1 according to the minima of energy.

Aiming to have a more complete analysis on the influence of the Gaussian’s curvature sign in the meron-like magnetization configuration lying on a curved surface, we have performed a numerical calculation of the energy’s minima for these magnetic structures in function of *c*. Main results are presented in Fig. 4, in which the behaviour of the meron’s exchange and total energy versus the continuous parameter *c* is plotted. In this figure, we have considered the chirality and polarity minimizing the energy of vortex and antivortex. The crossing of the curves associated with vortex (blue triangles) and antivortex (red circles) at *c* = 0 is promptly observed. That is, there is a curvature-induced selection of the winding number of merons in curved surfaces. Figure 4a also presents the energy of a configuration in which the magnetic moments point along the normal direction (blue asterisks).

Figure 4a also reveals the remarkable feature that there is the crossing in both sides of the plot between the normal magnetization field and the merons. Indeed, this effect occurs because, in this figure, only exchange energy is calculated. In fact, when the in-plane anisotropy term is considered, there is a qualitative change of this behaviour aside from the fact that the normal magnetization case is no longer a minimum energy configuration, as it is shown in Fig. 4b. Indeed, the inclusion of an in-plane anisotropy interaction leads to an increase in the energy of the normal magnetization, as well as the energy of merons (see Fig. 4b).

## Conclusions and Perspectives

In this work, we have determined the magnetic energy of curved surfaces in function of the Gaussian curvature’s sign. From using a model considering exchange and in-plane anisotropy, we have shown that the polarity of merons is intrinsically related to their chiralities. In addition, the global minimum energy occurs for vortex (antivortex) if the magnetic system has positive (negative) curvature.

We have also shown that the competition between exchange and in-plane anisotropy leads to the formation of a core on the meron configuration, but the presence of this core does not bring new qualitative changes in the global minimum energy obtained in function of the curvature.

Finally, we have presented a discussion on the minimum energy configuration lying on a curved surface by varying the curvature. It is shown that there is a crossing in the energy of vortex and antivortex meron-like configuration when *c* = 0, showing a curvature-induced selection of the meron’s winding number. This phenomenon is in agreement with previously obtained results for diagonal metrics, and it is a consequence of the DM-like interaction that appears in curved magnetic systems.

In the negative curvature case, there is a natural generalization of the fact that there is a link between the number of depressions and the winding number of the anti-vortex able to adapt to the surface. We must expect, for example, that in the monkey saddle case (a negative surface with three depressions) the anti-vortex minimizing the energy should be the one with winding number equal to −3. In this sense, the generalization of this work is obvious to be established but not easy to calculate and it exceeds the scope of this article.
